# Horses as athletes: the road to success

**DOI:** 10.1093/af/vfac024

**Published:** 2022-06-14

**Authors:** Sarah A Reed

**Affiliations:** Department of Animal Science, University of Connecticut, Storrs, CT,USA

From Xenophon and the beginning of classical equitation, through the Medieval Ages and use of the horse in battle, to the Renaissance and Haute Ecole, to our modern uses of horses in polo, racing, jumping, driving, pleasure, and more, the horse has been hailed as an athlete in its own right. Over time, as the demands of the use have changed, our horses have become more specialized and adapted to each new challenge. While there are many factors that influence a horse’s athletic ability, this issue will focus on some of the many physical characteristics that allow the horse to perform in a wide variety of disciplines.

The powerhouse of athletic movement is the muscle, where the mitochondria provide energy for muscle function. Christine Latham, Chloey Guy, Lauren Wesolowski, and Sarah White-Springer from Texas A&M University focus on the muscle’s mitochondrial phenotype, which is initially set by genetics and early development but can be refined by exercise training to improve performance (Latham et al., 2022). While much is still unknown about optimizing mitochondrial function, management, nutrition, and exercise training are promising options to improve performance.

Critical to mitochondrial energy production is the delivery of oxygen to the muscle. Respiratory capacity in equine athletes is far greater than other athletic species (Mazan, 2022). Melissa Mazan from Tufts University describes how, as obligate nose breathers with coupled stride and ventilation frequency at the gallop, horses also have respiratory limitations to overcome. Because of the high demands for oxygen, even minor impacts from upper and lower respiratory airway diseases can limit equine performance.

The skeletal system also has a critical role in equine performance, as it withstands the stressors placed on bones and joints during high-impact exercises. Raquel Baccarin, Sarah Seidel, Yara Michelacci, Paula Tokawa, and Tiago Oliviera from the University of São Paulo describe how osteoarthritis is common in equine athletes and, without proper diagnosis and treatment, can lead to prolonged joint pain and lameness (Baccarin et al., 2022). However, early recognition and treatment of joint degeneration can halt further degeneration and increase the chances of returning to performance.

Neural inputs coordinate the complex movements required of performance horses. Daniela Bedenice and Amy Johnson from Tufts University and University of Pennsylvania, respectively, focus on how mild signs of neurological conditions can mimic lameness conditions (Bedenice and Johnson, 2022). In particular, cervical vertebral stenotic myelopathy (Wobbler’s disease), equine degenerative myeloencephalopathy, and equine protozoal myeloencephalitis are three of the most common noncontagious neurological diseases in horses. Equine athletes with mild neurological conditions can often be treated and perform at low levels of competition but may have limited longevity as performance horses.

The biomechanics of the horse allow for graceful, powerful movements unique to their intended event. Sarah Jane Hobbs from the University of Central Lancashire and Hillary Clayton from Michigan State University describe the locomotor biomechanics that allow for high-speed galloping, show jumping, and the dressage movement, passage. In each, the horse is asked to perform movements that combine power, speed, balance, and grace that require coordinated efforts from the muscular, skeletal, and neural systems (Hobbs and Clayton, 2022).

In addition to the intrinsic properties that influence equine athletic performance, the horse–human dyad is fundamental to successful performance. Data from Russ Best at the Waikato Institute of Technology in New Zealand indicate that polo provides a unique opportunity to study the interactions between riders and horses, as a rider uses a specific string of ponies repeatedly during a given tournament, allowing for frequent measurements on that pairing. Current technologies can assess speed and distance traveled, heart rate of both the horse and rider, accelerometry, and use pressure monitoring to describe the points of contact between horse and rider (Best, 2022). 

Of further interest to many within and outside of the horse industry is what becomes of horses when their performance careers are finished. Ahern and Crawford (2022) describe the low incidence of musculoskeletal injuries (0.6% per week) and fatalities in Thoroughbred racehorses in Queensland, Australia. Interestingly, the risk of injury increased in horses that started training at later ages. Furthermore, 98% of horses in this study were retired and retrained for other careers, indicating that retired racing thoroughbreds can successfully adapt to other careers and maintain a lower level of athletic performance.

Horses are unique animals, with an incredible capacity for athletic performance. However, the line between maximal performance and injury is narrow and is easily crossed in the pursuit of improved performance. Perhaps, it is best to remember that “A horse gallops with his lungs, perseveres with his heart, and wins with his character.” (Federico Tesio).

## About the Author

**Sarah Reed** is an associate professor in the Department of Animal Science at the University of Connecticut. She earned her MS and PhD in Animal Science and then spent 2 yr as a post-doctoral research associate in the Department of Physical Therapy at the University of Florida. Her current research interests include the impacts of maternal diet during gestation on offspring growth and development, and the effects of exercise training and detraining in horses. She enjoys teaching, in particular introducing undergraduate students to research through class and research opportunities.

## References

[CIT0001] Ahern, B.J., and K.L. Crawford. 2022. Perspectives: Investigations into thoroughbred racehorse welfare in Queensland Australia focused on musculoskeletal injuries and retirement. Anim. Front. 12(3):59–62. https://doi.org/10.1093/af/vfac018 10.1093/af/vfac018 PMC9197301 35711502

[CIT0002] Baccarin, R., S. Seidel, Y. Michelacci, P. Towkawa, and T. Oliviera. 2022. Osteoarthritis: a common disease that should be avoided in the athletic horse’s life. Anim. Front. 12(3):25–36. https://doi.org/10.1093/af/vfac026 10.1093/af/vfac026 PMC9197312 35711506

[CIT0003] Bedenice, D., and A.L. Johnson. 2022. Neurologic conditions in the sport horse. Anim. Front. 12(3):37–44. https://doi.org/10.1093/af/vfac036 10.1093/af/vfac036 PMC9197298 35711509

[CIT0004] Best, R . 2022. Perspectives: The player–pony dyad in polo, lessons from other sports and future directions. Anim. Front. 12(3):54–58. https://doi.org/10.1093/af/vfac003 10.1093/af/vfac003 PMC9197297 35711510

[CIT0005] Hobbs, S.J., and H.M. Clayton. 2022. Citius, altius, fortius—communiter. The Olympic motto through the lens of equestrian sports. Anim. Front. 12(3):45–53. https://doi.org/10.1093/af/vfac025 10.1093/af/vfac025 PMC9197309 35711501

[CIT0006] Latham, C.M., C.P. Guy, L.T. Wesolowski, and S.H. White-Springer. 2022. Fueling equine performance: importance of mitochondrial phenotype in equine athletes. Anim. Front. 12(3):6–14. https://doi.org/10.1093/af/vfac023 10.1093/af/vfac023 PMC9197311 35711513

[CIT0007] Mazan, M . 2022. Equine exercise physiology—challenges to the respiratory system. Anim. Front. 12(3):15–24. https://doi.org/10.1093/af/vfac035 10.1093/af/vfac035 PMC9197307 35711503

